# Sound Richness of Music Might Be Mediated by Color Perception: A PET Study

**DOI:** 10.1155/2015/241804

**Published:** 2015-10-07

**Authors:** Masayuki Satoh, Ken Nagata, Hidekazu Tomimoto

**Affiliations:** ^1^Department of Dementia Prevention and Therapeutics, Graduate School of Medicine, Mie University, 2-174 Edobashi, Tsu, Mie 514-8507, Japan; ^2^Department of Neurology, Research Institute for Brain and Blood Vessels, 6-10 Senshu-Kubota-Machi, Akita 010-0874, Japan; ^3^Department of Neurology, Mie University Graduate School of Medicine, 2-174 Edobashi, Tsu, Mie 514-8507, Japan

## Abstract

*Objects*. We investigated the role of the fusiform cortex in music processing with the use of PET, focusing on the perception of sound richness. *Method*. Musically naïve subjects listened to familiar melodies with three kinds of accompaniments: (i) an accompaniment composed of only three basic chords (chord condition), (ii) a simple accompaniment typically used in traditional music text books in elementary school (simple condition), and (iii) an accompaniment with rich and flowery sounds composed by a professional composer (complex condition). Using a PET subtraction technique, we studied changes in regional cerebral blood flow (rCBF) in simple minus chord, complex minus simple, and complex minus chord conditions. *Results*. The simple minus chord, complex minus simple, and complex minus chord conditions regularly showed increases in rCBF at the posterior portion of the inferior temporal gyrus, including the LOC and fusiform gyrus. *Conclusions*. We may conclude that certain association cortices such as the LOC and the fusiform cortex may represent centers of multisensory integration, with foreground and background segregation occurring at the LOC level and the recognition of richness and floweriness of stimuli occurring in the fusiform cortex, both in terms of vision and audition.

## 1. Introduction

Historically, the style of music has developed from simple to complex. Such development was typically classified as a change from monophony, that is, music for a single voice or part [1], to polyphony, in which two or more strands sound simultaneously, or to homophony in which there was a clear distinction between melody and accompanying harmony [1]. In music with a monophonic style, only the melody is produced and there is no accompaniment. In homophony, to which most nursery and folk songs of western music belong, music consists of melody and its accompaniment. As music with homophonic or polyphonic styles has developed, harmonies have become more complex. For example, music of Mozart or Haydn in the 18th century rarely utilized dissonant chords, while the 20th century music of Ravel or Debussy had several kinds of chords including dissonant ones. Listening to homophonic music is different from listening to monophonic music according to the following. First, with homophonic music, listeners discriminate melody and its accompaniment. Even if the melody and the accompaniment are played by the same instrument (i.e., with the identical timbre), we can easily and instantaneously perceive the melody and the accompaniment. The neural basis of this is still unknown, but we previously reported in a positron emission tomography (PET) activation study that the lateral occipital complex (LOC), which participates in foreground and background segregation in vision, plays an important role in the discrimination between melody and its accompaniment [2]. The melody and the accompaniment could be regarded, in auditory terms, as the foreground and background, respectively. We suggested that the same neural substrates carried out similar functions beyond the simple discrimination of sensory modalities. Second, the sounds of homophonic music could be richer than monophonic music. The quality of sound is generally called “timbre.” The timbre is operationally defined as the attribute that distinguishes sounds of equal pitch, loudness, location, and duration [3]. The term “timbre” not only relates to the individual musical instrument, but also relates to expressing the characteristics of the sound of musical pieces. For example, it is generally considered that the timbre of impressionist music of Ravel or Debussy is richer and more flowery than the classical music of Mozart or Haydn. In the above-mentioned PET study, the melody with accompaniment also activated the fusiform cortex (in addition to the LOC) compared to the melody without the accompaniment [2]. We interpreted the activation of the fusiform cortex to reflect the rich sound from the accompaniment, but much still remains to be done to identify the role of that area in listening to music.

Over the past few decades, a considerable number of PET activation studies have been made on various aspects of music, sounds, and the brain [4–6], not only in healthy subjects [4, 6] but also in patients with tinnitus [5]. Based on our previous researches, we performed another PET study that investigated brain region activity while subjects listened to melodies with various kinds of accompaniments. Musically naïve subjects listened to melodies of familiar nursery songs with various degrees of sound richness of the accompaniment. According to a visual analogue scale (VAS), for each piece of music we also ascertained to what extent the subjects felt the sound was rich. Using a PET subtraction technique, brain regions that were significantly activated by sound richness were identified.

## 2. Subjects and Methods

### 2.1. Subjects

Ten right-handed male volunteers (mean age 21.7 ± 0.95 years; range 20–24) participated in the study. All were students at the Schools of Engineering or Mining, Akita University, and met criteria for Grison's second level of musical culture [7]. None had received any formal or private musical education, and none had any signs or history of neurological, cardiovascular, or psychiatric disease. All subjects gave written informed consent after the purpose and procedure of the examination had been fully explained. The study was approved by the Ethics Committee of the Research Institute for Brain and Blood Vessels, Akita, Japan, and all experiments were conducted in accordance with the Declaration of Helsinki.

### 2.2. Task Procedures

The stimuli in this experiment were six melodies of well-known Japanese nursery songs. All subjects were very familiar with these melodies. For each melody, the following three kinds of accompaniment were composed: (i) an accompaniment composed by using only three basic chords (tonic, dominant, and subdominant chord), one of which was set on each bar (chord condition), (ii) a simple accompaniment that is typically used in the traditional music text books in Japanese elementary schools (simple condition), and (iii) an accompaniment with rich and flowery sounds composed by a professional composer [8] (complex condition). The (i) chord and (ii) simple condition accompaniments were composed by one of the authors (Masayuki Satoh). The accompaniment of simple condition consisted of quarter tones of a chord on a whole note of fundamental tone. The first beat of each cord in the bar was rest, so only the fundamental tone was played at the first beat. All musical stimuli were played using the “FINALE” software [9]. The author Masayuki Satoh wrote musical scores of musical pieces used in this experiment on the “FINALE,” and the software played each piece with piano timbre. Each performance was recorded on a compact disc. Melodies with the three types of accompaniments were randomly presented. Subjects were instructed to listen to each melody, and PET measurements were obtained while listening to these melodies (procedures described below). Subjects were required to make a sign with the index finger of the right hand as the melody of each song finished. All stimuli were presented binaurally via inset stereo earphones.

The instruction to the subjects was as follows: Close your eyes. You will listen to a melody of a familiar nursery song. If you feel that the melody has finished, please make a sign with the index finger of your right hand.

### 2.3. Positron Emission Tomography Measurements

The protocol used in this study has been previously described in detail [2, 10–12]. Briefly, PET data were acquired in 3D acquisition mode using Headtome V (Shimadzu, Kyoto, Japan). Scans were performed in a darkened room with subjects lying supine with eyes closed. Nine CBF measurements were determined for each subject, three during the chord, three during the simple, and three during the complex condition. Employing ^15^O-labeled water (H_2_^15^O) intravenous bolus technique [13], emission data were collected for 90 seconds for each measurement following intravenous bolus injection of about 15 mL (40 mCi) H_2_^15^O. A musical piece was initiated 15 seconds prior to data acquisition, followed by another musical piece, and this in total continued for about 120 seconds. Emission data were corrected for attenuation by acquiring 10 minutes of transmission data utilizing ^68^Ge orbiting rod source performed prior to the activation scans. A wash-out period of approximately 10 minutes was allowed between successive scans. For anatomic reference, all subjects underwent axial T1-weighted imaging (T1WI) and T2-weighted imaging (T2WI) using a 1.5 T magnetic resonance system (Vision, Siemens, Germany). T1WI (TR/TE = 665/14 ms) and T2WI (TR/TE = 3600/96 ms) were obtained using a slice thickness of 5 mm with an interslice gap of 1 mm.

### 2.4. Data Analysis

PET data analysis was performed on a SGI Indy running IRIX 6.5 (Silicon Graphics, California), using an automated PET activation analysis package [14] composed of six main processing stages which has been previously described in detail [2, 10–12]. The six main stages consisted of intrasubject coregistration, intrasubject normalization, automatic detection of the AC-PC line, detection of multiple stretching points and surface landmarks on intrasubject averaged image sets, intersubject summation and statistical analyses, and superimposition of statistical results onto the stereotactic MRI. Deformation of individual brains to correspond with the standard atlas brain was achieved by spatially matching individual landmarks to the corresponding predefined standard surface landmarks and minimizing correlation coefficients of regional profile curves between the stretching centers. Activation foci were considered to be significantly activated if the corresponding *p* value was less than a predetermined threshold (*p* < 0.001, Bonferroni correction for multiple comparisons). Anatomical identification of activation foci was achieved by referring the stereotactic coordinates of the peak activated pixels to the standard Talairach brain atlas [15].

### 2.5. Visual Analogue Scale (VAS) of Sound Richness

**Figure 1:**
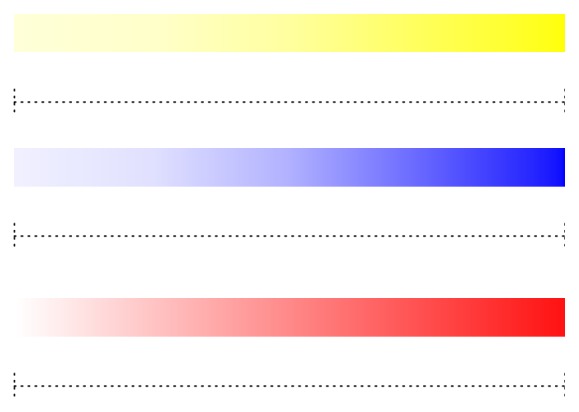
Visual analogue scale (VAS) for the assessment of subjective impression of sound richness of each musical stimulus. The length of color bar is 140 mm.

After the PET measurement, the degree of sound richness of each melody with the three types of accompaniments was investigated in each subject. In a quiet room, each subject listened to the stimuli and was required to subjectively mark the VAS (Figure 1) according to the degree of sound richness the subject felt. Three colors (yellow, blue, and red) were used because the lyrics of some songs had a relationship with a specific color, for example, the sea related to blue and the sunset to red. Subjects marked to the right to the degree that they felt that the sound of the music was rich. We measured the distance from the left end to the marked position (mm) and, using the Wilcoxon signed rank test, statistically compared the distance between the three kinds of accompaniments, namely, chord, simple, and complex condition.

## 3. Results

**Figure 2:**
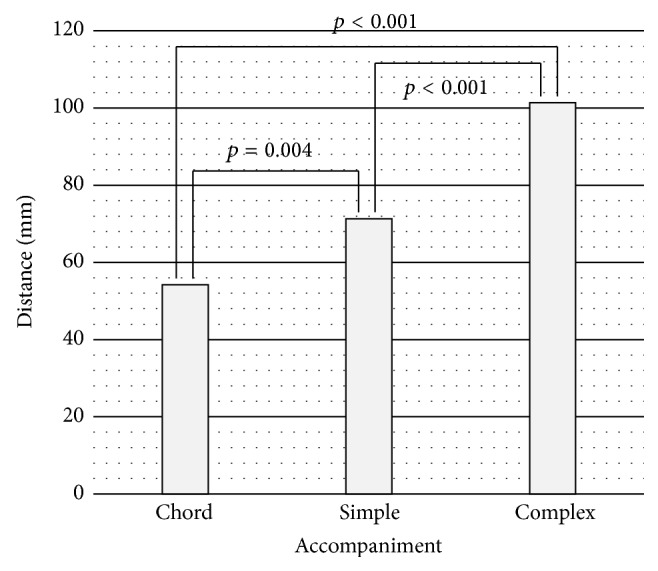
Results of VAS of each accompaniment condition. As the accompaniment became more complex, the subjects regarded the sound of musical pieces as being richer.

Regarding the VAS of sound richness, the mean distance from the left end was significantly longer as the accompaniment became more complex (Figure 2): chord condition 54.2 ± 34.2; simple condition 71.3 ± 30.1; complex condition 101.4 ± 31.1 mm (mean ± standard deviation (sd)). We can reasonably conclude that, as expected, the more complex the accompaniment became, the richer the subjects reported the sound.

**Figure 3:**
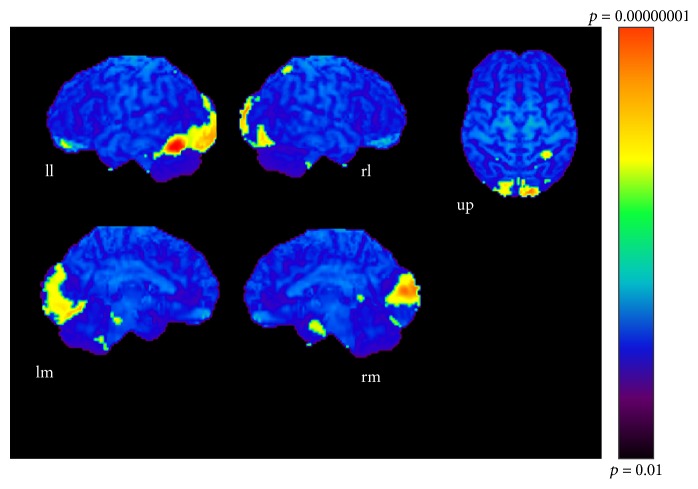
Simple-chord condition (*p* < 0.001). ll: lateral surface of left hemisphere; lm: medial surface of left hemisphere; rl: lateral surface of right hemisphere; rm: medial surface of right hemisphere; up: upper surface.

**Figure 4:**
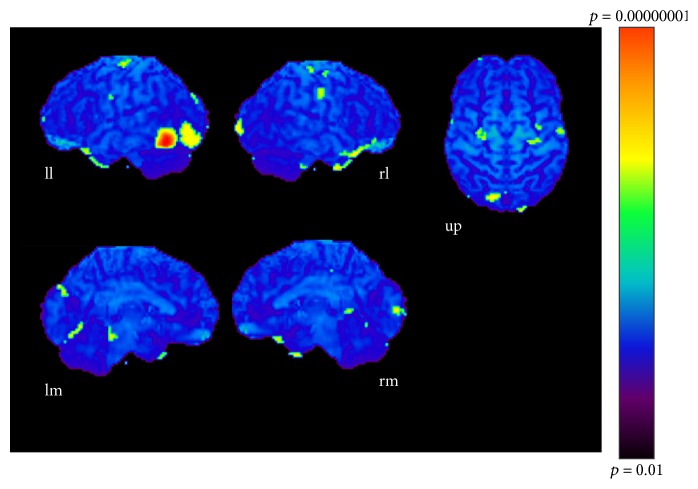
Complex-chord condition (*p* < 0.001). ll: lateral surface of left hemisphere; lm: medial surface of left hemisphere; rl: lateral surface of right hemisphere; rm: medial surface of right hemisphere; up: upper surface.

**Figure 5:**
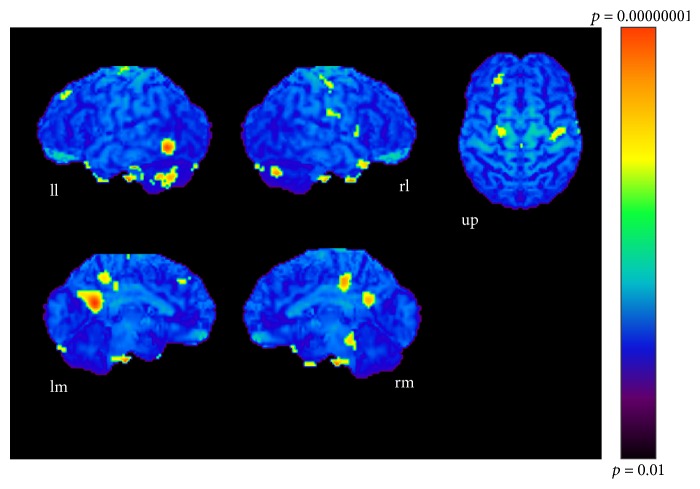
Complex-simple condition (*p* < 0.001). ll: lateral surface of left hemisphere; lm: medial surface of left hemisphere; rl: lateral surface of right hemisphere; rm: medial surface of right hemisphere; up: upper surface.

**Figure 6:**
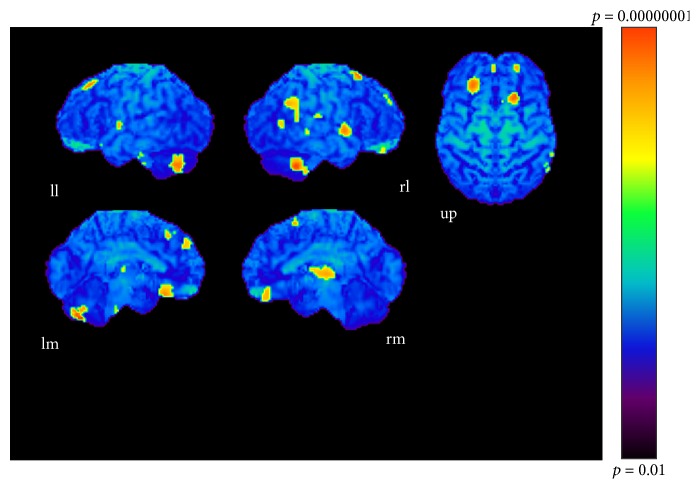
Chord-simple condition (*p* < 0.001). ll: lateral surface of left hemisphere; lm: medial surface of left hemisphere; rl: lateral surface of right hemisphere; rm: medial surface of right hemisphere; up: upper surface.

**Figure 7 fig7:**
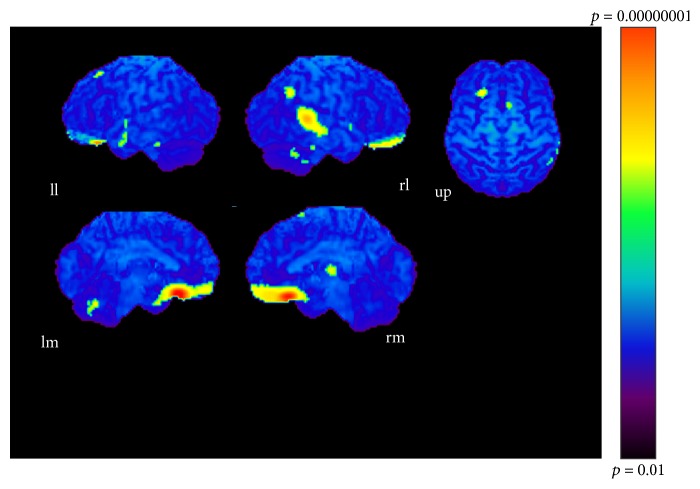
Chord-complex condition (*p* < 0.001). ll: lateral surface of left hemisphere; lm: medial surface of left hemisphere; rl: lateral surface of right hemisphere; rm: medial surface of right hemisphere; up: upper surface.

**Figure 8:**
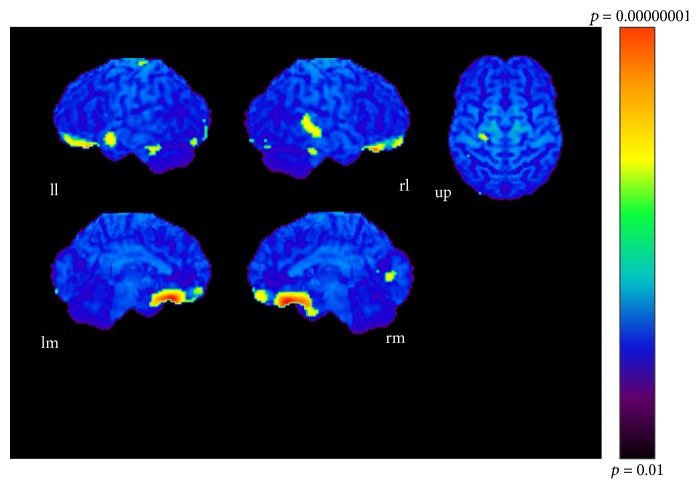
Simple-complex condition (*p* < 0.001). ll: lateral surface of left hemisphere; lm: medial surface of left hemisphere; rl: lateral surface of right hemisphere; rm: medial surface of right hemisphere; up: upper surface.

The results of subtractions providing significant regions activated as the sound became more complex are given in Tables 1, 2, and 3 and Figures 3, 4, and 5. The regions activated during the simple condition but not during the chord condition are listed in Table 1 together with stereotactic coordinates based on the brain atlas of Talairach and Tournoux [15]. These results show areas of relative blood flow changes that emphasize differences between the two conditions and minimize areas that are common to both conditions. Significant increases in relative cortical blood flow were found in the posterior portion of the left inferior temporal gyrus, bilateral fusiform gyri, the medial surface of the bilateral frontal lobes, the right superior parietal lobule, and the left orbital frontal cortex (Table 1, Figure 3). Compared to the chord condition, the complex condition produced significant activation at the posterior portion of the left inferior temporal gyrus, left fusiform gyrus, right medial surface of the occipital lobe, the lateral surface of the left occipital lobe, and the anterior portion of the left middle temporal gyrus (Table 2, Figure 4). Between the complex and simple condition, the former condition significantly activated the posterior portion of the left inferior temporal gyrus, the left fusiform gurus, the left retrosplenial region, the anterior portion of the right middle temporal gyrus, the right cingulate gyrus, and the bilateral cerebellum (Table 3, Figure 5). The important point to note is that the activation of the posterior portion of the inferior temporal gyrus and the fusiform gyrus was observed in all results after every subtraction, that is, simple minus chord, complex minus chord, and complex minus simple condition. The opposite subtraction of chord minus simple, chord minus complex, and simple minus complex conditions revealed almost the same activation pattern. The activation was observed at the bilateral orbital frontal cortex, the bilateral or left superior frontal gyrus, and the right superior temporal gyrus (Tables 4 [Table tab5]–6, Figures 6 [Fig fig7]–8).

## 4. Discussion

The findings of this experiment are summarized as follows: as an accompaniment became more complex, (i) the subjects felt that the sound of music was richer and (ii) the fusiform cortex and the posterior portion of the inferior temporal gyrus were activated. In the following paragraphs, we discuss the functional significance of these activated brain regions.

The fusiform cortex might participate in the perception of sound richness. The present study showed that, as the sound became richer, the activation of the fusiform cortex increased. This finding revealed that the degree of the activation of the fusiform cortex was different depending on the degree of the sound richness of the accompaniment in the identical melodies. It is generally accepted that the fusiform cortex processes color recognition, based on the results of a case [16] and a PET activation study [17]. The findings of the present study and previous reports suggest that color information in vision and sound richness in audition might be similarly registered in the brain. In other words, it is possible that similar information from different sensory modalities might be processed within the same brain region and that the visual association cortex might not only be involved in visual processing. Recent studies have revealed that some sensory modalities are related to each other. This phenomenon is called “cross-modal integration” and was observed between taste and audition [18], taste and smell [19–22], taste and color [23], odor and color [24], taste and music [25], pitch and visual size [26, 27], brightness and frequency of vibrotactile stimuli [28], sound and color [29, 30], and vision and audition [31]. It was reported that cross-modal associations are ubiquitously present in normal mental function [25, 32, 33]. Recent research suggests that cortical auditory processing is divided into separate processing streams [31, 34]. Posterior temporoparietal regions, labeled the “where” or “how” stream, may be specialized for processing sound motion and location [31]. Regions anterior and ventral to primary auditory cortex, labeled the “what” stream, may be specialized for processing characteristic auditory features [31]. Neurons in “what” stream respond directly to auditory and visual sensory stimuli and are important for forming the association between auditory and visual objects [31]. Therefore, we may conclude that cross-modal integration also occurs at the fusiform cortex between color and sound richness when listening to music.

**Figure 9:**
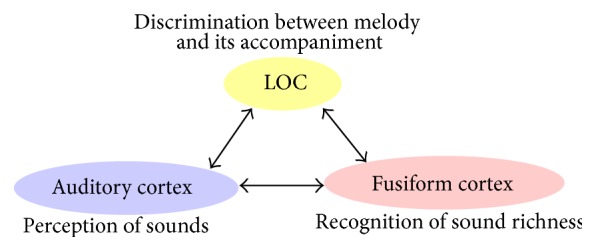
Diagram of cognitive processing during listening to music with accompaniment.

In the present study, the posterior portion of the inferior temporal gyrus was also activated. This area is called the lateral occipital complex (LOC) and is known to participate in foreground and background segregation in vision [35]. It was suggested that the LOC also participates in the discrimination between melody and its accompaniment [2]. In our previous study, we considered that the LOC might play a similar role of foreground and background segregation in both vision and audition. This finding reinforced the hypothesis that some association cortices carry out a similar function beyond the differences in sensory modalities (Figure 9). After the perception of sounds at the auditory cortex level, the information might be sent to the LOC and fusiform cortex. The former and the latter might participate in the foreground and background segregation and the recognition of sound richness, respectively, both in vision and audition.

The opposite subtraction, namely, chord minus simple, chord minus complex, and simple minus complex condition, all produced an activation of the bilateral orbital frontal cortex. The functional significance of this region in this experiment is unclear. However, this region is known as a structure within Yakovlev's circuit that participates in emotion and memory. Damage to this region often results in disinhibition, impairment in control over impulsive behavior based on instinct and emotion. It is possible that activation of the orbital frontal cortex was caused by the comfortable and pleasant feeling of listening to familiar nursery songs or by inhibiting the desire to sing along with these familiar melodies.

In summary, the fusiform cortex and the LOC might have a similar function in vision and audition. The fusiform cortex recognizes color and sound richness, and the LOC participates in foreground and background segregation. We may conclude that the association cortices might play a similar role across multiple sensory modalities. Further studies are needed to clarify the multimodal integration of association cortices.

## Supplementary Material

Examples of auditory stimuli of chord, simple, and complex condition of Japanese nursery song “Scene of Winter”.

## Figures and Tables

**Table 1:** Regions showing significant changes in rCBF by the subtraction of simple minus chord condition.

Anatomical structures	Brodmann area	Talairach coordinate			*z*-score
		*x*	*y*	*z*	
Posterior portion of inferior temporal gyrus	37				
L		−48	−55	−16	5.20
Fusiform gyrus					
L	18/19	−19	−58	−9	4.38
R	19	30	−67	−7	4.32
Medial surface of occipital lobe	17/18				
L		−17	−87	−4	3.83
R		10	−87	11	4.024
Superior parietal lobule	7				
R		33	−53	56	3.32
Orbital frontal cortex	11				
L		−17	50	−16	3.08

Coordinates *x*, *y*, and *z* are in millimetres corresponding to the atlas of Talairach and Tournoux. The *x*-coordinate refers to medial-lateral position relative to midline (negative = left); *y*-coordinate refers to anterior-posterior position relative to the anterior commissure (positive = anterior); *z*-coordinate refers to superior-inferior position relative to the anterior commissure-posterior commissure line (positive = superior). *z*-score refers to the maximum pixel of the region. L and R refer to the left and right hemisphere, respectively.

**Table 2:** Regions showing significant changes in rCBF by the subtraction of complex minus chord condition.

Anatomical structures	Brodmann area	Talairach coordinate			*z*-score
		*x*	*y*	*z*	
Posterior portion of inferior temporal gyrus	37				
L		−53	−58	−11	4.97
Fusiform gyrus					
L	19/37	−30	−49	−11	3.58
Medial surface of occipital lobe					
R	17	17	−96	2	3.10
Lateral surface of occipital lobe					
L	18	−39	−73	−2	3.00
Anterior portion of middle temporal gyrus					
R	38	35	8	−40	3.96

Details as for Table 1.

**Table 3:** Regions showing significant changes in rCBF by the subtraction of complex minus simple condition.

Anatomical structures	Brodmann area	Talairach coordinate			*z*-score
		*x*	*y*	*z*	
Posterior portion of inferior temporal gyrus	37				
L		−60	−58	−7	3.81
Fusiform gyrus					
L	36	−33	−26	−25	4.27
Retrosplenial region					
L	29	−6	−51	18	3.91
Anterior portion of middle temporal gyrus					
R	38	37	8	−40	3.44
Cingulate gyrus					
R	31	10	−28	40	3.29
Cerebellum					
L		−51	−49	−38	3.34
R		39	−64	−32	3.34

Details as for Table 1.

**Table 4:** Regions showing significant changes in rCBF by the subtraction of chord minus simple condition.

Anatomical structures	Brodmann area	Talairach coordinate			*z*-score
		*x*	*y*	*z*	
Orbital frontal cortex	11				
L		−6	26	−16	2.97
R		5	24	−14	2.73
Superior frontal gyrus	6/8				
L		−24	30	43	3.13
R		21	19	58	3.29
Superior temporal gyrus					
R	22	51	3	2	3.06
Cerebellum					
L		−17	−62	−36	3.82
R		51	−46	−36	3.43

Details as for Table 1.

**Table 5 tab5:** Regions showing significant changes in rCBF by the subtraction of chord minus complex condition.

Anatomical structures	Brodmann area	Talairach coordinate			*z*-score
		*x*	*y*	*z*	
Orbital frontal cortex	10/11				
L		−3	26	−18	5.39
R		12	64	−11	3.20
Superior frontal gyrus					
L	8	−24	28	50	3.05
Superior temporal gyrus					
R	22	62	−37	7	3.75

Details as for Table 1.

**Table 6:** Regions showing significant changes in rCBF by the subtraction of simple minus complex condition 3.

Anatomical structures	Brodmann area	Talairach coordinate			*z*-score
		*x*	*y*	*z*	
Orbital frontal cortex	11				
L		−1	28	−20	4.79
R		12	32	−18	4.06
Anterolateral portion of superior frontal gyrus					
L	21/22	−48	5	−14	2.97
Superior temporal gyrus					
R	22	62	−37	7	2.90
Cerebellum					
L		−51	−37	−25	3.17

Details as for Table 1.
